# MutationTaster2021

**DOI:** 10.1093/nar/gkab266

**Published:** 2021-04-24

**Authors:** Robin Steinhaus, Sebastian Proft, Markus Schuelke, David N Cooper, Jana Marie Schwarz, Dominik Seelow

**Affiliations:** Berliner Institut für Gesundheitsforschung in der Charité – Universitätsmedizin Berlin, 10117 Berlin, Germany; Institut für Medizinische Genetik und Humangenetik, Charité – Universitätsmedizin Berlin, corporate member of Freie Universität Berlin and Humboldt-Universität zu Berlin, 13353 Berlin, Germany; Berliner Institut für Gesundheitsforschung in der Charité – Universitätsmedizin Berlin, 10117 Berlin, Germany; Institut für Medizinische Genetik und Humangenetik, Charité – Universitätsmedizin Berlin, corporate member of Freie Universität Berlin and Humboldt-Universität zu Berlin, 13353 Berlin, Germany; Klinik für Pädiatrie m.S. Neurologie, Charité – Universitätsmedizin Berlin, corporate member of Freie Universität Berlin and Humboldt-Universität zu Berlin, 13353 Berlin, Germany; NeuroCure Clinical Research Center, Charité – Universitätsmedizin Berlin, corporate member of Freie Universität Berlin and Humboldt-Universität zu Berlin, 10117 Berlin, Germany; Institute of Medical Genetics, School of Medicine, Cardiff University, Cardiff, CF14 4XW, UK; Klinik für Pädiatrie m.S. Neurologie, Charité – Universitätsmedizin Berlin, corporate member of Freie Universität Berlin and Humboldt-Universität zu Berlin, 13353 Berlin, Germany; Berliner Institut für Gesundheitsforschung in der Charité – Universitätsmedizin Berlin, 10117 Berlin, Germany; Institut für Medizinische Genetik und Humangenetik, Charité – Universitätsmedizin Berlin, corporate member of Freie Universität Berlin and Humboldt-Universität zu Berlin, 13353 Berlin, Germany

## Abstract

Here we present an update to MutationTaster, our DNA variant effect prediction tool. The new version uses a different prediction model and attains higher accuracy than its predecessor, especially for rare benign variants. In addition, we have integrated many sources of data that only became available after the last release (such as gnomAD and ExAC pLI scores) and changed the splice site prediction model. To more easily assess the relevance of detected known disease mutations to the clinical phenotype of the patient, MutationTaster now provides information on the diseases they cause. Further changes represent a major overhaul of the interfaces to increase user-friendliness whilst many changes under the hood have been designed to accelerate the processing of uploaded VCF files. We also offer an API for the rapid automated query of smaller numbers of variants from within other software. MutationTaster2021 integrates our disease mutation search engine, MutationDistiller, to prioritise variants from VCF files using the patient's clinical phenotype. The novel version is available at https://www.genecascade.org/MutationTaster2021/. This website is free and open to all users and there is no login requirement.

## Graphical Abstract

**Graphical Abstract:**
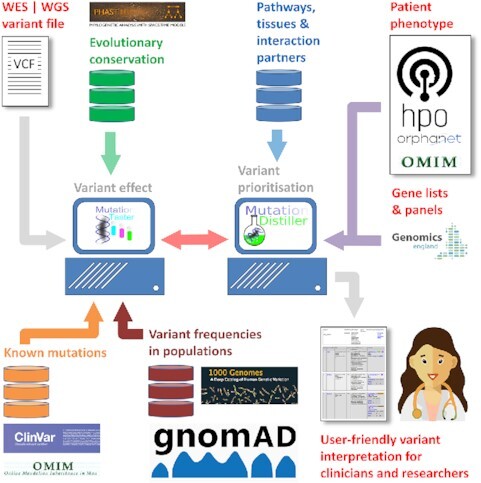
Identification of disease-causing variants with MutationTaster2021.

## INTRODUCTION

The last decade has witnessed a huge increase in the number of reported disease mutations causing monogenic disorders (1). Whereas in the past the inheritance of disease-linked regions was studied by linkage analysis, and positional and functional candidate genes were then sequenced for promising variants, high-throughput sequencing has completely changed the picture. With the advent of Whole Exome Sequencing (WES), the complete coding sequence of an individual can be readily sequenced, ensuring the capture of virtually all coding sequence mutations. Consequently, linkage information is no longer required, and biomedical researchers can focus on those variants that are likely to have a deleterious effect on genes with a role in disease pathogenesis. Whilst this strategy permits the identification of disease mutations within single individuals without any other cases or family members (2,3), the usual approach is to compare genotypes in different affected or unaffected family members (4).

Even though a battery of variant effect prediction tools is now available, e.g. PolyPhen-2 (5), SIFT (6), MutationTaster (7) or CADD (8), none of these tools reaches an accuracy much above 90%. Thus, with tens of thousands of DNA variants detected in any given WES run, thousands of potentially deleterious variants remain to be assessed. Filtering against known polymorphisms found in large-scale sequencing projects such as the 1000 Genomes Project (9), ExAC (10), or gnomAD (11) (which are all integrated in MutationTaster2021) further reduces the number of possible disease-causing variants. However, because many polymorphisms are population-specific, only a portion of the benign variants can be removed using this strategy. In this context, the use of dedicated phenotype-aware disease mutation search engines such as eXtasy (12), the Exomiser (13) or MutationDistiller (14) can play a role. These combine the predicted deleteriousness of a given variant with the potential of a particular ‘mutated’ gene to play a role in the disease. Although this approach has been successful in many cases, in general only about one third of WES studies lead to a molecular diagnosis (15,16).

With the latest release of MutationTaster, we hope to increase the overall success rate for WES. As with its predecessor, MutationTaster2021 subjects each variant to a battery of *in silico* tests. With the aim of increasing prediction accuracy, we have replaced the Bayes classifier with Random Forest models tailored to different types of variants. When training these models, we focused on the balanced accuracy, i.e. the same prediction quality for benign and deleterious variants. This significantly improved the prediction results (see Results). We have also worked on the user-friendliness of MutationTaster and integrated MutationDistiller for the identification of disease mutations causing monogenic disorders.

## CHANGES IN THE NEW VERSION

### Training with ‘rare’ variants

#### Benign variants

For the new version, we strove to reduce the number of false positive predictions. Even though the filtering against common polymorphisms greatly reduced the false positive rate, many rare or population-specific variants remained as false positives. The most likely explanation for this is that these false positives display a much higher degree of phylogenetic conservation than the frequent polymorphisms (at least 20 healthy homozygous individuals) we had previously used as benign training data. To circumvent this, we employed as benign training cases all intragenic variants from gnomAD (11) for which there was at least one homozygous carrier.

#### Deleterious variants

As in the previous version, the deleterious training cases comprise intragenic disease mutations from the Professional Version of the Human Gene Mutation Database (HGMD^®^ Pro) (1) and from ClinVar (17). We restricted our analysis to those variants labelled as ‘DM’ in HGMD Pro or ‘pathogenic’ or ‘likely pathogenic’ in ClinVar. We excluded ClinVar variants with conflicting labels.

Variants that were found in both training sets (deleterious and benign) were excluded. Our final training sets consisted of 11 168 768 benign and 236 400 deleterious variants (see Supplementary Table S1).

### New models for UTR variants

To provide better predictions for variants in the untranslated regions (UTRs), we set up dedicated prediction models for the two UTRs. Although these have lower accuracies than the model for other non-coding variants, they still provide better results for UTR variants. MutationTaster2021 now uses five dedicated models for variants (i) causing single amino acid substitutions, (ii) causing more than one amino acid substitution, (iii) located in the 5′ UTR, (iv) located in the 3′ UTR and (v) all other intragenic variants (see Supplementary Table S2).

### New classifier: Random Forest instead of Naive Bayes

Whereas the previous releases of MutationTaster (7,18) used a Naive Bayes classifier to make predictions, we have shifted the prediction to Random Forest models so as to improve the results. The number of features and the number of trees were separately optimised for each of the five models. Details of the predictive performance of the different models are given in Supplementary Table S2. Supplementary Table S3 shows the performance of the previous version, MutationTaster2. The implementation of the classifiers is described in the Supplement. Instead of the internal probability of the Bayes classifier used in the previous releases, the new output indicates how many decision trees of the Random Forest are suggestive of deleteriousness.

We have attempted to find a reasonable trade-off between predictive performance and speed and therefore limited tree number and tree size within the different Random Forest models. A grid search showed that in two prediction models, Random Forests with only one third of the size of the ‘perfect forest’ could be used without losing >0.12% balanced accuracy. Detailed information about the forests that were trained and tested can be found on our website and in the Supplement. The characteristics of the Random Forests used by MutationTaster2021 are listed in Supplementary Table S4.

It should be noted that these predictors were explicitly trained for balanced accuracy, i.e. the same predictive performance for benign and deleterious variants. Although this increases the number of false positive predictions, it decreases the risk of missing a real disease mutation compared to predictors trained for specificity.

### Variant effect on splicing

In the past, MutationTaster has used nnsplice (19) for the prediction of the effect of variants on splicing. In internal tests, we found that MaxEntScan (20) showed higher accuracy and we therefore switched to MaxEntScan. This came with another advantage: MaxEntScan was written in Perl and could easily be transferred into a Perl module which can be run under mod_perl (see Supplement) thereby bringing enormous speed gains. One drawback of MaxEntScan is however that it is limited to variants in canonical splice sites.

It should be noted that MutationTaster2 and MutationTaster2021 do not search for cryptic splice sites activated by DNA variants as this was found to yield too many false positive predictions.

### Integration of gnomAD, ExAC and ExAC pLI scores

Since the last publication of MutationTaster, several new data sources have been integrated (also in MutationTaster2). Most notable are the genotype counts from ExAC (10) and gnomAD (11) for the exclusion of variants also found in healthy individuals, and ExAC pLI scores to assess whether a gene is tolerant of loss-of-function variants. We have dropped the use of HapMap (21) SNP genotypes.

Whilst homozygous individuals from the 1000 Genomes Project (9), ExAC and gnomAD are used to automatically classify variants as benign, the pLI scores are not used to classify variants.

All other data sources used by MutationTaster have been updated in the new release; external data sources and software are listed in Supplementary Tables S5 and S6.

### New interfaces

The most striking difference with respect to the previous releases are the overhauled interfaces. We attempted to preserve the old layout to keep MutationTaster recognisable while giving it a more modern appearance. We also streamlined the input interfaces and restructured the output to make the most relevant options or results more prominent.

When variants are listed in ClinVar as either ‘pathogenic’ or ‘likely pathogenic’, this information and the disease caused by the variant are displayed in the output (see Supplementary Figure S2). Variants clearly assigned as ‘pathogenic’ in ClinVar are automatically labelled as deleterious by MutationTaster.

The three different modes (analysis of a single variant using a transcript/CDS position, analysis of a variant using its physical position, and analysis of complete VCF files) are now offered in the same interface and their names make their purpose much clearer (see Supplementary Figure S1).

### VCF analysis pipeline

Whilst the analysis pipeline for VCF files was called ‘QueryEngine’ in the last release, it is now more intuitively termed ‘VCF analysis pipeline’ and comes with clearer interfaces. An invisible change is a complete restructuring of the underlying database structure. The prediction results of variants are now permanently stored and re-used whenever the same variant is queried again. This has a dramatic effect on the speed of the application, with a typical WES VCF file now being analysed in <5 min.

The contents of single VCF files are automatically removed after 4 weeks but can be actively deleted by the uploaders. The variant results stored in the database do not contain any information about their origin, i.e. it is impossible to reconstruct a VCF file once it has been deleted.

The changes in the database structure also allow the parallel use of different versions of MutationTaster built for different genome versions (not yet implemented) and different releases of Ensembl (22).

### API

MutationTaster2021 offers an API for the automatic query of several variants from within other tools, e.g. VarFish (23). For fast prediction times, the results of such queries are not stored in our database and have to be recalculated for each new call. We have therefore restricted the API use to 50 variants in one call and instead encourage our users to upload VCF files for larger sets of variants – predictions made in the VCF analysis pipeline are stored in the database.

Use of the API is described at https://www.genecascade.org/MutationTaster2021/info/#api.

### Integration of MutationDistiller

In addition to the previously offered options for sorting and filtering the results of the VCF analysis pipeline, MutationTaster2021 tightly integrates our disease mutation search engine MutationDistiller (14). With MutationDistiller, users can easily prioritise potential disease-causing variants in terms of the biological role of the affected genes. Users can of course also download MutationTaster's predictions to employ them in any application they so choose.

## RESULTS

**Figure 1.**
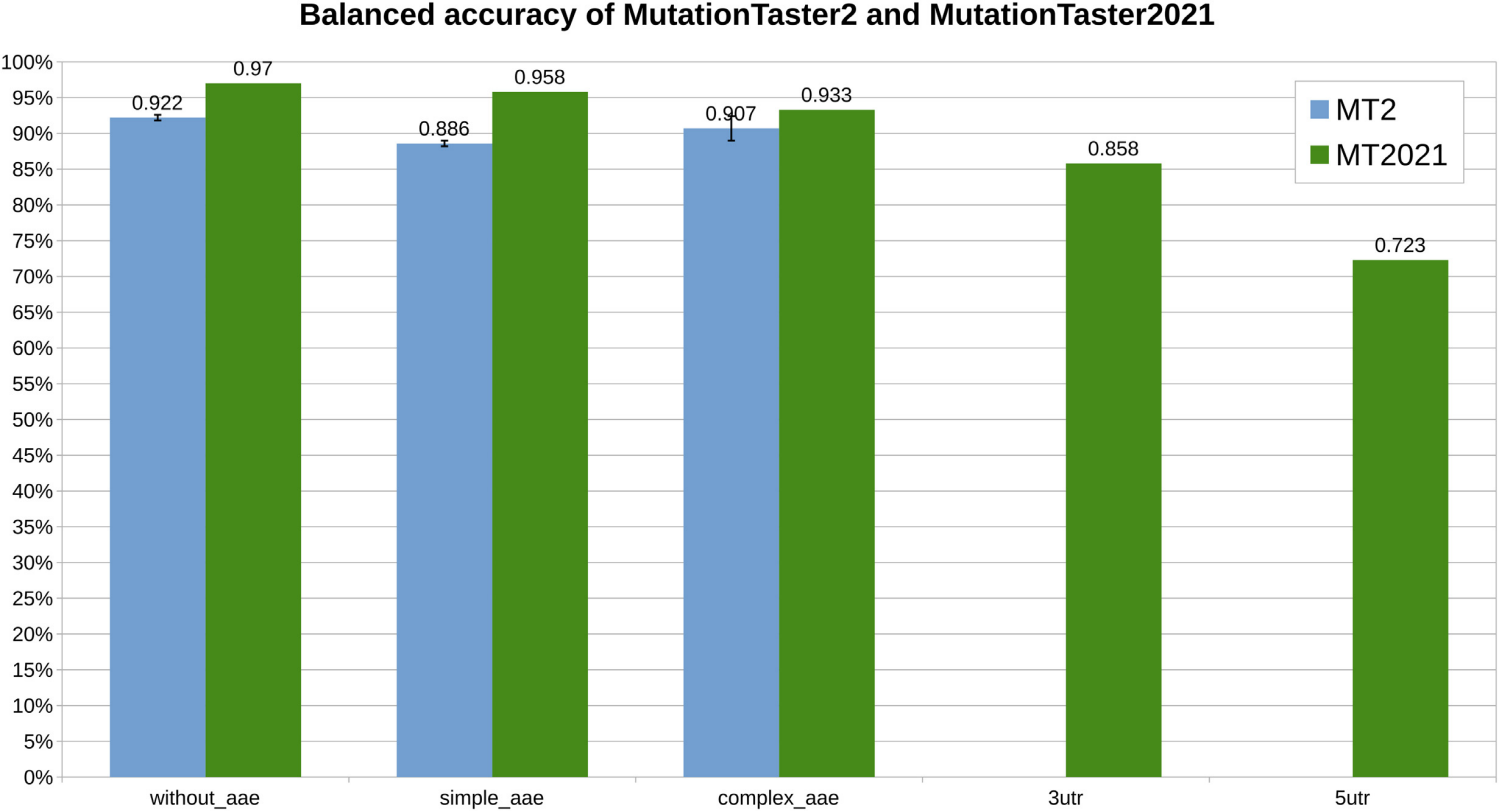
Balanced accuracy of MutationTaster2 and MutationTaster2021. Figure 1 shows the gains in the balanced accuracy of MutationTaster2021 (green) compared to MutationTaster2 (blue) for the five different prediction models (*simple_aae*: variants leading to single amino acid substitutions; *complex_aae*: variants changing more than one amino acid; *3utr*: variants in the 3′ UTR; *5utr*: variants in the 5′ UTR; *without_aae*: all other variants, i.e. non-coding variants). The models for UTR variants were not available in MutationTaster2. Please note that the accuracy of MutationTaster2 was validated in five-fold cross-validations whereas the accuracy of MutationTaster2021 was measured on data not used for training. A thorough description and detailed statistics can be found in the Supplement.

The changes in the new version allow a much faster and more accurate prediction of the effect of DNA variants. The Random Forest classifiers increase the balanced accuracy from 92.2% to 97.0% for non-coding variants, from 88.6% to 95.8% for variants causing single amino acid substitutions, and from 90.7% to 93.3% for variants causing more significant changes in the amino acid sequence (e.g. small deletions, frameshifts or premature stop codons). We have generated new models for variants in the UTRs; however, these yield balanced accuracies of only 85.8% (3′ UTR) or 72.3% (5′ UTR). A comparison of the balanced accuracy of both versions is depicted in Figure 1.

In addition, the analysis has become much more rapid due to the caching of already-made predictions and we have overworked the user interfaces for a clearer workflow.

## DISCUSSION

MutationTaster is explicitly aimed at biomedical researchers who want to identify the pathological mutation(s) in a patient suffering from a suspected monogenic disease. Unlike other tools such as CADD, we provide a binary prediction (deleterious or benign) and present the information associated with a given variant (e.g. evolutionary conservation or altered splicing) in a user-readable interface. Although there was no linear correlation between the deleteriousness and the probability given by the Bayes classifier in the old version, the use of small Random Forests renders such a correlation impossible. For this reason, we no longer give a potentially misleading confidence score but instead print the number of decision trees supporting deleteriousness versus benign. As with any classifier, a number of variants will be misclassified. This becomes especially apparent for benign variants (tens of thousands in any WES). As mentioned earlier, our models were explicitly trained for balanced accuracy, not for low false positive rates (specificity). This follows our patient-oriented maxim that it is better to err on the side of generating some false positives than to run the risk of missing a true positive variant, i.e. the actual disease-causing mutation. MutationTaster uses data from different large-scale genotyping projects to automatically exclude common variants but we recommend using in-house databases to filter out population-specific polymorphisms.

To save disk space, we do not store the exact numbers of heterozygous or homozygous genotypes in these databases but instead cut off numbers at 32 000 as this number of healthy individuals clearly indicates benign alleles. We provide direct links to the original data should our users require exact numbers.

With biomedical researchers in mind as the main users of MutationTaster, we hope to streamline their search for causal variants in their patients with the seamless integration of MutationDistiller as a downstream application. Thus, MutationTaster/MutationDistiller can serve as a ‘one-stop shop’ for the identification of disease mutations.

One clear limitation of MutationTaster is that it can only predict the deleteriousness of variants residing within protein-coding genes. Variants within RNA genes or outwith genes cannot be assessed. Although we offer some prediction functions for such variants in our tool RegulationSpotter (24), we should state that we do not consider predictions for extragenic variants as reliable enough to be included in MutationTaster.

## OUTLOOK

### Use of genome version 38

So far, MutationTaster only uses human genome version 37 (GRCh37), the predominant version used by the medical genetics community. A major goal for the next year will be the inclusion of the current genome version, GRCh38. The new database structure facilitates the parallel use of different genome versions.

### Splicing

We are currently evaluating the accuracies of various splice prediction tools and may replace MaxEntScan with a tool with higher predictive performance in the near future.

### Better predictions for mtDNA variants

We also aim to improve the prediction of the effects of variants in the mitochondrial DNA. This will include predictions for variants within the tRNA genes (which cannot yet be analyzed) and handling of the different annotations of the mitochondrial genome (chrM versus chrMT).

### Better predictions for variants located in the untranslated regions

The low predictive performance for variants located in the UTRs will be improved in the near future. So far, the changes to other non-coding variants include the use of polyadq (25) to detect disrupted polyadenylation signals (3′ UTR) and a self-made check for changes to the Kozak consensus sequence (5′ UTR). Obviously, both models will benefit from adding further tools to improve the recognition of relevant changes in the DNA, e.g. to predict changes in mRNA stability.

### Handling of multi-sample VCF files

The current release of MutationTaster is limited to VCF files containing a single sample. We are already working on the extension to multi-sample VCF files.

## DATA AVAILABILITY

MutationTaster2021 is freely available at https://www.genecascade.org/MutationTaster2021/. We provide examples, concise documentation, and detailed information about the Random Forest classifier. The website is free and open to all users and there is no login requirement.

The old release is still available at http://www.mutationtaster.org/ (please note that some novel functions such as ExAC genotype counts, pLI scores, and the display of diseases have already been implemented).

## Supplementary Material

gkab266_Supplemental_File
