# Supplementary material for: MutationTaster2021
Source: Nucleic Acids Res. 2021 Apr 24;49(W1):W446–51. doi: 10.1093/nar/gkab266 (PMC8262698; doi:10.1093/nar/gkab266)
Supplement: gkab266_Supplemental_File [file gkab266_supplemental_file.pdf]

MutationTaster2021

Supplement

Robin Steinhaus[1,2], Sebastian Proft[1,2] , Markus Schuelke[3,4], David N. Cooper[5], Jana Marie Schwarz[3], and Dominik Seelow[1,2,*]

[1] Berliner Institut für Gesundheitsforschung in der Charité – Universitätsmedizin Berlin, Berlin, 10117, Germany

[2] Institut für Medizinische Genetik und Humangenetik, Charité – Universitätsmedizin Berlin, corporate member of Freie Universität Berlin and Humboldt-Universität zu Berlin, Berlin, 10117, Germany

[3] Klinik für Pädiatrie m.S. Neurologie, Charité – Universitätsmedizin Berlin, corporate member of Freie Universität Berlin and Humboldt-Universität zu Berlin, Berlin, 10117, Germany

[4] NeuroCore Clinical Research Center, Charité – Universitätsmedizin Berlin, corporate member of Freie Universität Berlin and Humboldt-Universität zu Berlin, Berlin, 10117, Germany

[5] Institute of Medical Genetics, School of Medicine, Cardiff University, Cardiff, CF14 4XW, UK

* To whom correspondence should be addressed. Tel: +49 30 450 543684; Fax: +49 30 7543906; Email: dominik.seelow@charite.de

SUPPLEMENTARY TABLES

Supplementary Table S1. Variants used to train the classification models

Model	Benign [n]	Deleterious [n]	
**variants**	11168768	236400	all variants
**without_aae**	30984304	81843	non-coding
**simple_aae**	507702	270634	affecting a single amino acid
**complex_aae**	32432	349660	affecting more than one amino acid
**3utr**	415995	816	located in the 3' UTR
**5utr**	112176	923	located in the 5' UTR

This table shows the number of cases used to train the five different models used by MutationTaster2021. It should be noted that due to the existence of multiple transcripts, a variant can lead to more than one training case. For variants that could be assigned to either the *simple_aae* or the *complex_aae* model, all non-coding cases (i.e. intron locations in other transcripts) were removed.

Supplementary Table S2. Predictive performance of MutationTaster2021

	without_aae	simple_aae	complex_aae	3utr	5utr
**Training cases [n]**	23299610	583752	286569	312608	84824
**Test cases [n]**	7766537	194584	95523	104203	28275
**Deleterious test cases [n]**	20461	67658	87415	204	231
**NPV**	0.999	0.966	0.975	0.999	0.995
**PPV (precision)**	0.979	0.962	0.988	0.980	0.990
**Sensitivity (recall)**	0.939	0.935	0.998	0.716	0.446
**Specificity**	0.999	0.980	0.868	0.999	0.999
**Balanced accuracy**	0.970	0.958	0.933	0.858	0.723

Presented is the predictive performance of MutationTaster2021 for the five different models. Results were obtained with test cases that were not used for training (NPV: negative predictive value, PPV: positive predictive value).

The actual performance of MutationTaster2021 is even better, as common polymorphisms and known disease mutations are automatically detected and categorised.

Supplementary Table S3. Predictive performance of MutationTaster2

	without_aae	simple_aae	complex_aae
**NPV**	0.957 [0.003]	0.877 [0.008]	0.869 [0.032]
**PPV (precision)**	0.888 [0.006]	0.895 [0.005]	0.944 [0.004]
**Sensitivity (recall)**	0.954 [0.004]	0.879 [0.007]	0.879 [0.026]
**Specificity**	0.895 [0.005]	0.893 [0.004]	0.939 [0.005]
**Balanced accuracy**	0.922 [0.004]	0.886 [0.004]	0.907 [0.017]

This table depicts the results of the cross-validation of the three different models of MutationTaster2 (standard deviation in brackets).

Supplementary Table S4. Characteristics of the Random Forest models

Model	without_aae	simple_aae	complex_aae	3utr	5utr
**Training [n]**	23299610	583752	286569	312608	84824
**RF trees [n]**	100	100	200	100	300
**Split criterion**	entropy	entropy	gini	gini	entropy
**AUC-ROC**	0.990 [<0.001]	0.987 [<0.001]	0.993 [<0.001]	0.942 [0.015]	0.893 [0.008]
**Balanced accuracy**	0.959 [0.001]	0.940 [<0.001]	0.907 [0.002]	0.826 [0.014]	0.709 [0.007]

These are characteristics of the different Random Forest (RF) models used in the five prediction models, determined in a grid search to find the best models (see **Random Forest model selection** in the Methods part below for a description). Area under the curve / receiver operating characteristics (AUC-ROC) and balanced accuracy were measured in a threefold cross-validation of the complete data set, standard deviation in brackets.

Supplementary Table S5. External data sources used by MutationTaster2021

Software	Description
Ensembl(1)	general genetic data
NCBI Entrez(2)	
dbSNP(2)	dbSNP IDs of known variants
ClinVar(3)	pathogenicity and disease reports for variants
HGMD public(4)	position of public disease mutations from HGMD
1000 Genomes Project(5)	genotype counts in in healthy controls
ExAC(6)	
gnomAD(7)	
PhastCons(8)	phylogenetic conservation (DNA level)
PhyloP(9)	
UniProt(10)	protein domains

Supplementary Table S6. External software used by MutationTaster2021

Software	Description
blast2(11)	conservation at protein level
MaxEntScan(12)	splice site prediction
polyadq(13)	test for poly-adenylation signals

SUPPLEMENTARY FIGURES

Supplementary Figure S1.   New landing page

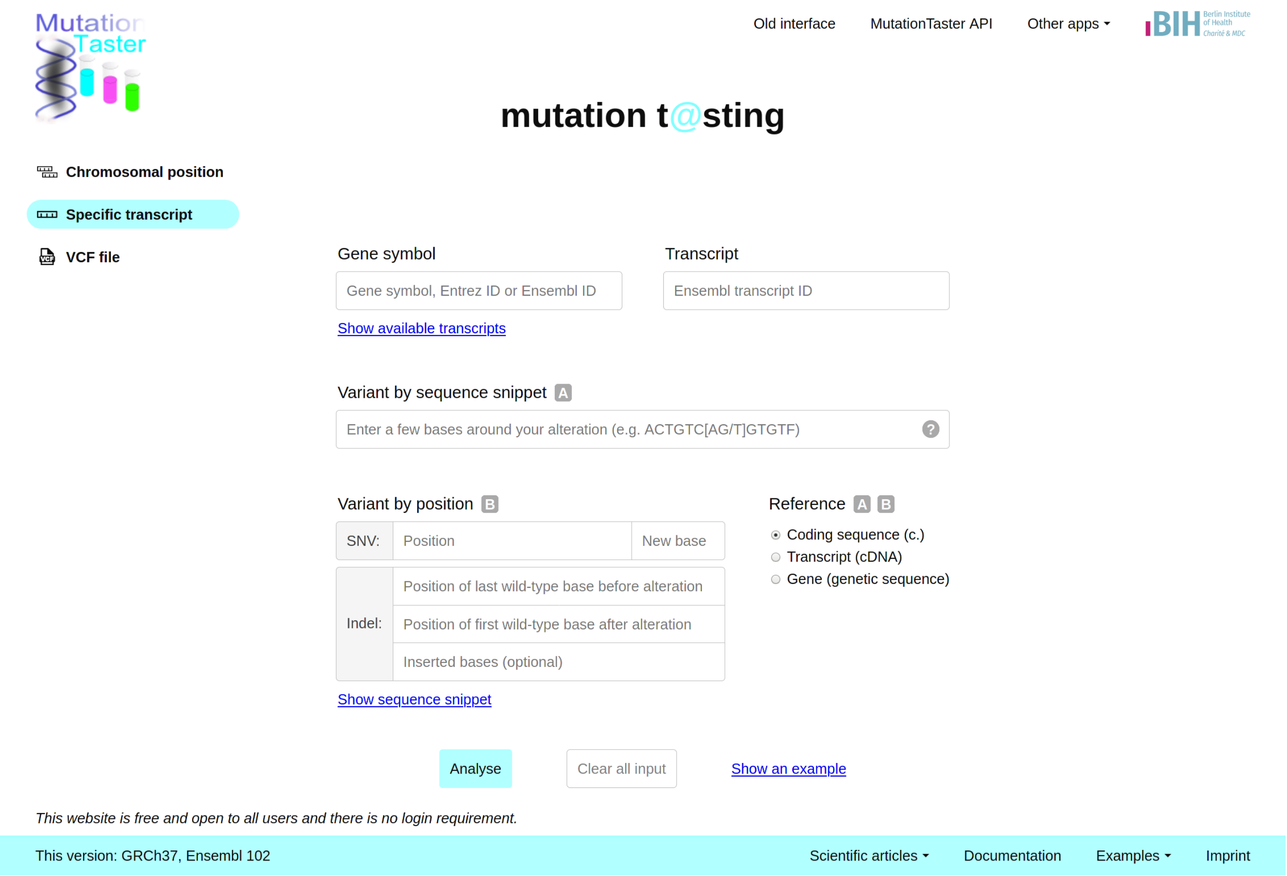

Landing page of MutationTaster2021. The three different modes (analysis of a variant based on its physical position, analysis of a single variant based on a transcript/CDS position, and analysis of complete VCF files) are now shown in the same interface.

https://www.genecascade.org/MutationTaster2021/

Supplementary Figure S2. MT2021 Results for a known disease mutation

MutationTaster

mutation t@sting

Prediction: Deleterious — Permalink

Summary:
- **Amino acid sequence changed**
- **Known disease mutation at this position (HGMD CM081556)**
- **Known disease mutation: ClinVar ID 18371 (pathogenic)**
- **Protein features (might be) affected**
- Model: simple_aae
- Tree vote: 95|5 (del | benign)
- Automatic classification due to ClinVar

Analysed issue	Analysis result
Phys. location	chr2:233391374T>C show variant in all transcripts IGV
Gene symbol	CHRND
ExAC LOF metrics	LOF: 0.00, misssense: 0.60, synonymous: 0.19
Ensembl transcript ID	ENST00000258385
Genbank transcript ID	
UniProt peptide	Q07001
Variant type	Single base exchange
Gene region	CDS
DNA changes	c.188T>C<br>g.672T>C
AA changes	L63P Score: 98<br>Explain score(s)
Frameshift	No
Length of protein	Normal
Known variant	Allele 'C' was neither found in ExAC, 1000G nor gnomAD.<br>**Known disease mutation:** ClinVar variation ID 18371 (**pathogenic for *Congenital myasthenic syndrome 3B***)<br>OMIM<br>Known disease mutation at this position, please check HGMD for details (HGMD ID CM081556)
Phylogenetic conservation	*PhyloP* *PhastCons*<br>(flanking) 4.101 1<br>4.743 1<br>(flanking) 1.009 1<br>Explain score(s) and/or inspect your position(s) in in UCSC Genome Browser
Splice sites	No abrogation of potential splice sites
Distance from splice site	11
Kozak consensus sequence altered?	No
poly(A) signal	N/A
Protein conservation	*Species* / *Match* / *Gene* / *AA* / *Alignment*<br>Human / / / 63 / VDVALALTLSNLISLKEVEETLTT<br>mutated / not conserved / / 63 / SNPISLKEVEETLT<br>Ptroglodytes / all identical / ENSPTRG00000013040 / 63 / SNLISLKEVEETLT<br>Mmulatta / all identical / ENSMMUG00000022147 / 76 / SNLISLKEVEETLT<br>Fcatus / all identical / ENSFCAG00000030943 / 63 / SNLISLKEVEETLT<br>Mmusculus / all identical / ENSMUSG00000026251 / 66 / LTLSNLISLKEVEETLT<br>Ggallus / all identical / ENSGALG00000007899 / 63 / VDVYLALTLSNLISLK<br>Trubripes / no alignment / ENSTRUG00000026623 / n/a /<br>Drerio / all identical / ENSDARG00000019342 / 63 / VDIYLALTLSNLISLKEVDETLL<br>Dmelanogaster / no homologue<br>Celegans / no homologue<br>Xtropicalis / all identical / ENSXETG00000027884 / 63 / VNVSLALTLSNLISLKEADETLT
Protein features	*Start (aa)* *End (aa)* *Feature* *Details*<br>22 245 TOPO_DOM Extracellular lost
AA sequence altered	Yes
Chromosome	2
Strand	1
Original gDNA sequence snippet	GGCCCTCACACTCTCCAACCTCATCTCCCTGGTGAGAGGCC
Altered gDNA sequence snippet	GGCCCTCACACTCTCCAACCCCATCTCCCTGGTGAGAGGCC
Original cDNA sequence snippet	GGCCCTCACACTCTCCAACCTCATCTCCCTGAAAGAAGTTG
Altered cDNA sequence snippet	GGCCCTCACACTCTCCAACCCCATCTCCCTGAAAGAAGTTG
Wildtype AA sequence	MEGPVLTLGL LAALAVCGSW GLNEEERLIR HLFQEKGYNK ELRPVAHKEE SVDVALALTL<br>SNLISLKEVE ETLTTNVWIE HGWTDNRLKW NAEEFGNISV LRLPPDMVWL PEIVLENNND<br>GSFQISYSCN VLVYHYGFVY WLPPAIFRSS CPISVTYFPF DWQNCSLKFS SLKYTAKEIT<br>LSLKQDAKEN RTYPVEWIII DPEGFTENGE WEIVHRPARV NVDPRAPLDS PSRQDITFYL<br>IIRRKPLFYI INILVPCVLI SFMVNLVFYL PADSGEKTSV AISVLLAQSV FLLLISKRLP<br>ATSMAIPLIG KFLLFGMVLV TMVVVICVIV LNIHFRTPST HVLSEGVKKL FLETLPELLH<br>MSRPAEDGPS PGALVRRSSS LGYISKAEEY FLLKSRSDLM FEKQSERHGL ARRLTTARRP<br>PASSEQAQQE LFNELKPAVD GANFIVNHMR DQNNYNEEKD SWNRVARTVD RLCLFVVTPV<br>MVVGTAWIFL QGVYNQPPPQ PFPGDPYSYN VQDKRFI*
Mutated AA sequence	MEGPVLTLGL LAALAVCGSW GLNEEERLIR HLFQEKGYNK ELRPVAHKEE SVDVALALTL<br>SNPISLKEVE ETLTTNVWIE HGWTDNRLKW NAEEFGNISV LRLPPDMVWL PEIVLENNND<br>GSFQISYSCN VLVYHYGFVY WLPPAIFRSS CPISVTYFPF DWQNCSLKFS SLKYTAKEIT<br>LSLKQDAKEN RTYPVEWIII DPEGFTENGE WEIVHRPARV NVDPRAPLDS PSRQDITFYL<br>IIRRKPLFYI INILVPCVLI SFMVNLVFYL PADSGEKTSV AISVLLAQSV FLLLISKRLP<br>ATSMAIPLIG KFLLFGMVLV TMVVVICVIV LNIHFRTPST HVLSEGVKKL FLETLPELLH<br>MSRPAEDGPS PGALVRRSSS LGYISKAEEY FLLKSRSDLM FEKQSERHGL ARRLTTARRP<br>PASSEQAQQE LFNELKPAVD GANFIVNHMR DQNNYNEEKD SWNRVARTVD RLCLFVVTPV<br>MVVGTAWIFL QGVYNQPPPQ PFPGDPYSYN VQDKRFI*
Position of stopcodon in wt / mu CDS	1554 / 1554
Position (AA) of stopcodon in wt / mu AA sequence	518 / 518
Position of stopcodon in wt / mu cDNA	1586 / 1586
Position of start ATG in wt / mu cDNA	33 / 33
Last intron/exon boundary	1403
Theoretical NMD boundary in CDS	1320
Length of CDS	1554
Coding sequence (CDS) position	188
cDNA position	220
gDNA position	672
Chromosomal position	233391374
Speed	0.16 s

All positions are in basepairs (bp) if not explicitly stated differently. cDNA/gDNA/chromosomal position: Ins/del are shown as 'last normal base / first normal base'.
AA/aa: amino acid; CDS: coding sequence; mu: mutated; NMD: nonsense-mediated mRNA decay; nt: nucleotide; wt: wildtype; TGP: 1000 Genomes Project

The SNV chr2:233391374T>C (GRCh37) in the *CHRND* gene is listed in NCBI ClinVar as a known disease-causing variant for *Myasthenic syndrome*.

METHODS

Selection of variants

Benign variants were selected from the gnomAD genotype repository (version 2.1.1). We considered all intragenic variants found in at least one individual in the homozygous state as benign. Variants without any allele frequency specifications were discarded.

We obtained deleterious intragenic variants from ClinVar (version 2020-12-08) and HGMD Pro (Version 2020Q03). ClinVar variants were included when they were annotated as 'pathogenic' or as 'likely pathogenic'; variants with other or conflicting labels were excluded. HGMD variants were used when they were labelled as 'DM' (disease mutation).

Variants found in both training sets were removed. The training data comprise single nucleotide variants as well as small insertions/deletions.

Selection of training cases

All variants were sent to MutationTaster. The results of MutationTaster's analyses were saved in dedicated database tables. These results comprised information such as outcome (deleterious vs. benign), affected transcripts, pre-mRNA localisation of the variant, conservation at the protein and DNA level and many more (see **Supplementary Table S5** for the data sources and **Supplementary Table S6** for the external software). A complete list of the features can be found at https://www.genecascade.org/downloads/MutationTaster2021/SupplementaryData/.

Depending on effect and pre-mRNA localisation of the variant within a transcript, the variant:transcript pair was assigned to the suitable model (see **Supplementary Table S1**).

Data pre-processing for the classification

The steps listed below were used to train the classifier but are also used for the classification of variants within MutationTaster2021.

Changes in the amino acid sequence

A variant can cause one (*simple_aae* model) or more (*complex_aae*) changes to the amino acid sequence. In the Random Forest models, each observed amino acid substitution in the whole training data set (including insertions, deletions, or nonsense variants, e.g. 'AP', '-A', 'A-' or 'A*') is treated as a single feature. In the *simple_aae* model, only one of these features can be true for a single variant; in the *complex_aae* model many features can be true.

phyloP / phastCons

We use the phyloP and phastCons values to reflect the phylogenetic conservation of a variant. In addition to the position at the variant site(s) itself, we assess the conservation at both flanking bases. Whilst the latter always contains two values per variant and metric (phyloP and phastCons), the variant sites may have multiple values in case of deletions of more than a single base.

After trying different models, we determined that using four different attributes (mean phyloP score of flanking bases, mean phastCons score of flanking bases, mean phyloP score of affected bases, mean phastCons score of affected bases) yielded the highest accuracy.

Protein features

Each variant can hit one or more functional domains in the protein. Our training data includes a column for each feature that could be lost due to the mutation (e.g. DISULFID). The entries in these columns are binary and specify whether the feature has been lost at least once for each variant.

Splicing

We prepared two features to handle the effect of a variant on splicing, "splice_quot_A" and “splice_quot_D”. These scores are calculated as the absolute ratio of the absolute difference between the wild-type score and the mutation score (mt) with the wild-type score (wt) for the acceptor and donor site, respectively, e.g. for a donor site:

splice_quot_D=abs(abs(mt_D-wt_D)/wt-D)

If the first/last base of an exon or the first/last two intronic bases are changed, we consider a splice site as lost and set the splice_quot to 10. If there is no effect of a variant on a nearby splice site, then the score is set to 0 instead.

It should be noted that MutationTaster2021 does not search for activated cryptic splice sites but only predicts the effect on known splice sites..

Dichotomisation

All other categorical (non-numeric or non-binary) attributes in the data were dichotomised to obtain features with a binary value (true/false).

Random Forest models

Feature removal

For each model, we separately checked the training data and removed columns and entries with no information. First, we removed all columns that did not contain any entries for any of the variants for that model. We then removed the columns with identical values for all variants.

In addition to the feature removal, we also removed training cases containing at least one column without any value.

Model generation and selection

We used the Python sklearn package to generate Random Forest models. We decided to train our models for the highest balanced accuracy as a trade-off between specificity (low false positive rate) and sensitivity (correct identification of disease-causing variants).

We started with the default parameters and performed a grid search for each of the five models to find the optimal hyperparameters for the number of trees used in each Random Forest and the criteria to find the optimal split for each of the nodes within each tree (either gini index or entropy), which are measures of impurity or information gain of a node in the tree. A detailed list of the combinations tested and the description of the Random Forest development are provided at

https://www.genecascade.org/downloads/MutationTaster2021/SupplementaryData/.

To avoid overfitting, we performed 3-fold cross-validations for each of our five models to select the best parameters for the number of trees in the Random Forest and the best criterion for determining the optimal split at each node. For this purpose, we randomly extracted 25% of our training data to withhold for the final performance test, while ensuring that the extracted samples followed the same distribution of positive and negative samples as present in the entire dataset. We trained the classifier on the remaining variants. The models were trained within a grid search, with possible hyperparameters set so that the number of trees within the Random Forest was either 100, 200, or 300 and the criterion for splits could be either the gini index or entropy. Three validation cycles were performed for each model (see **Supplementary Table S4** for the characteristics of the chosen models).

We additionally decided to not only select for models with a high predictive performance, but also for run-time performance, i.e. for small models. Therefore, we opted to pick the models with only 100 trees for *simple_aae* and *without_aae*. This decision resulted in a marginal decrease of balanced accuracy of 0.12% (*simple_aae*) and 0.05% (*without_aae*) compared to the 'perfect models', whilst leading to a size reduction of 67.1% (*simple_aae*) and 68.0% (*without_aae*), giving us an equivalent boost in classification speed. Final performance was then calculated on the test data set (see **Supplementary Table S2**). The classification uses the weighted prediction of the result leaf of each Random Forest tree, i.e. the fraction of deleterious cases *vs.* all cases for leaves predicting deleteriousness. Please note that most leaves give a binary result (i.e. all cases left are either benign or deleterious). The trees are available on our website.

We were thus able to improve the accuracy in all classification models, with a drastic increase in the *simple_aae* model (MutationTaster2 88.6%, MutationTaster2021 95.8%) and substantial changes in the *without_aae* model from 92.2% to 97.0% and in the *complex_aae* model from 90.7% to 93.3% (see **Supplementary Table S3** for MutationTaster2's performance).

IMPLEMENTATION

Data are stored in a PostgreSQL database. MutationTaster2021 is programmed in Perl and runs under mod_perl in an Apache web server. All user interfaces are written in HTML (with JavaScript and AJAX functions) and are developed for the Firefox browser under Linux, Mac OS, and Microsoft Windows and are regularly tested with Google Chrome, Safari and Microsoft Edge. We employ TORQUE (version 4.2) as job scheduling software. External tools used by MutationTaster2021 (MaxEntScan(12), bl2seq(11), polyadq(13)) run on a RAM disk to increase speed.

The Random Forests were trained in Python 3.6.12 using scikit-learn 0.23.2 and numpy 1.17.3, data preprocessing was done using pandas 1.1.5. Plots (downloadable from our website) were created using matplotlib 3.2.2. We used a Perl script to transform the Random Forest models into Perl data structures which can be accessed by the MutationTaster2021 software.

REFERENCES

1. Howe,K.L., Achuthan,P., Allen,J., Allen,J., Alvarez-Jarreta,J., Amode,M.R., Armean,I.M., Azov,A.G., Bennett,R., Bhai,J., *et al.* (2021) Ensembl 2021. *Nucleic Acids Res.*, **49**, D884–D891.
https://doi.org/10.1093/nar/gkaa942
http://www.ncbi.nlm.nih.gov/pmc/articles/PMC7778975

2. Sayers,E.W., Beck,J., Bolton,E.E., Bourexis,D., Brister,J.R., Canese,K., Comeau,D.C., Funk,K., Kim,S., Klimke,W., *et al.* (2021) Database resources of the National Center for Biotechnology Information. *Nucleic Acids Res.*, **49**, D10–D17.
https://doi.org/10.1093/nar/gkaa892
http://www.ncbi.nlm.nih.gov/pmc/articles/PMC7778943

3. Landrum,M.J., Chitipiralla,S., Brown,G.R., Chen,C., Gu,B., Hart,J., Hoffman,D., Jang,W., Kaur,K., Liu,C., *et al.* (2020) ClinVar: improvements to accessing data. *Nucleic Acids Res.*, **48**, D835–D844.
https://doi.org/10.1093/nar/gkz972
http://www.ncbi.nlm.nih.gov/pmc/articles/PMC6943040

4. Stenson,P.D., Mort,M., Ball,E.V., Chapman,M., Evans,K., Azevedo,L., Hayden,M., Heywood,S., Millar,D.S., Phillips,A.D., *et al.* (2020) The Human Gene Mutation Database (HGMD®): optimizing its use in a clinical diagnostic or research setting. *Hum. Genet.*, **139**, 1197–1207.
https://doi.org/10.1007/s00439-020-02199-3
http://www.ncbi.nlm.nih.gov/pmc/articles/PMC7497289

5. 1000 Genomes Project Consortium, Auton,A., Brooks,L.D., Durbin,R.M., Garrison,E.P., Kang,H.M., Korbel,J.O., Marchini,J.L., McCarthy,S., McVean,G.A., *et al.* (2015) A global reference for human genetic variation. *Nature*, **526**, 68–74.
https://doi.org/10.1038/nature15393
http://www.ncbi.nlm.nih.gov/pmc/articles/PMC4750478

6. Lek,M., Karczewski,K.J., Minikel,E.V., Samocha,K.E., Banks,E., Fennell,T., O'Donnell-Luria,A.H., Ware,J.S., Hill,A.J., Cummings,B.B., *et al.* (2016) Analysis of protein-coding genetic variation in 60,706 humans. *Nature*, **536**, 285–291.
https://doi.org/10.1038/nature19057
http://www.ncbi.nlm.nih.gov/pmc/articles/PMC5018207

7. Karczewski,K.J., Francioli,L.C., Tiao,G., Cummings,B.B., Alföldi,J., Wang,Q., Collins,R.L., Laricchia,K.M., Ganna,A., Birnbaum,D.P., *et al.* (2020) The mutational constraint spectrum quantified from variation in 141,456 humans. *Nature*, **581**, 434–443.
https://doi.org/10.1038/s41586-020-2308-7
http://www.ncbi.nlm.nih.gov/pmc/articles/PMC7334197

8. Siepel,A., Bejerano,G., Pedersen,J.S., Hinrichs,A.S., Hou,M., Rosenbloom,K., Clawson,H., Spieth,J., Hillier,L.W., Richards,S., *et al.* (2005) Evolutionarily conserved elements in vertebrate, insect, worm, and yeast genomes. *Genome Res.*, **15**, 1034–1050.
https://doi.org/10.1101/gr.3715005
http://www.ncbi.nlm.nih.gov/pmc/articles/PMC1182216

9. Pollard,K.S., Hubisz,M.J., Rosenbloom,K.R. and Siepel,A. (2010) Detection of nonneutral substitution rates on mammalian phylogenies. *Genome Res.*, **20**, 110–121.
https://doi.org/10.1101/gr.097857.109
http://www.ncbi.nlm.nih.gov/pmc/articles/PMC2798823

10. UniProt Consortium (2021) UniProt: the universal protein knowledgebase in 2021. *Nucleic Acids Res.*, **49**, D480–D489.
https://doi.org/10.1093/nar/gkaa1100
http://www.ncbi.nlm.nih.gov/pmc/articles/PMC7778908

11. Tatusova,T.A. and Madden,T.L. (1999) BLAST 2 Sequences, a new tool for comparing protein and nucleotide sequences. *FEMS Microbiol. Lett.*, **174**, 247–250.
https://doi.org/10.1111/j.1574-6968.1999.tb13575.x
http://www.ncbi.nlm.nih.gov/pubmed/10339815

12. Yeo,G. and Burge,C.B. (2004) Maximum entropy modeling of short sequence motifs with applications to RNA splicing signals. *J. Comput. Biol. J. Comput. Mol. Cell Biol.*, **11**, 377–394.
https://doi.org/10.1089/1066527041410418
http://www.ncbi.nlm.nih.gov/pubmed/15285897

13. Tabaska,J.E. and Zhang,M.Q. (1999) Detection of polyadenylation signals in human DNA sequences. *Gene*, **231**, 77–86.
https://doi.org/10.1016/s0378-1119(99)00104-3
http://www.ncbi.nlm.nih.gov/pubmed/10231571
